# Humic acid degradation by the synthesized flower-like Ag/ZnO nanostructure as an efficient photocatalyst

**DOI:** 10.1186/s40201-014-0138-y

**Published:** 2014-12-09

**Authors:** Mohammad Taghi Ghaneian, Pouran Morovati, Mohammad Hassan Ehrampoush, Masoumeh Tabatabaee

**Affiliations:** Department of Environmental Health Engineering, Shahid Sadoughi University of Medical Sciences, Yazd, Iran; Department of Chemistry, Yazd Branch, Islamic Azad University, Yazd, Iran

**Keywords:** Photocatalyst, Nano Ag/ZnO, Humic Acid, Degradation, Solar irradiation

## Abstract

Nano-sized flower-like Ag/ZnO was synthesized by a simple method using zinc acetate and silver acetate under hydrothermal condition. Powder X-ray diffraction (PXRD) and transmission electron microscopy (TEM) were used to characterize the structure and morphology of the synthesized powder. Nano flower-like Ag/ZnO was used as a photocatalyst for degradation of humic acid in aqueous solution. The disappearance of HA was analyzed by measuring the absorbance of sample at special wavelength (254 nm). The effects of various parameters such as amount of photocatalyst, pH, initial humic acid concentration and irradiation time on degradation rate were systematically investigated. Photodegradation efficiency was small when the photolysis was carried out in the absence of Ag/ZnO and it was also negligible in the absence of light. Approximately 70% of humic acid (50 mg/L) has been eliminated after 40 minutes in the presences of catalyst (catalyst dose o.6 g/L and pH =7) and UV_A_ irradiation. While, 100% of humic acid has been eliminated with solar irradiation.

## Introduction

Natural organic matters or NOMs, refers to a group of carbon-based compounds that are found in the surface water and some groundwater supplies. Humic substances represent a major fraction of natural organic matters. Humic substances are divided to three fractions: insoluble components in all pH values (humin); humic acid which is soluble in water at pH >2 and fulvic acids (FA), which are soluble in aqueous solutions at all pH values [1,2]. Macromolecus ofhumic acid have a backbone of aromatic and aliphatic residues with numerous substituent such as phenolic, ketenes, amino acids and carboxylic groups and carrying negative charges in natural waters [3-5]. Removal of humic acids is necessary before drinking water chlorination, due to the reaction between chlorine and humic acids in water treatment cause generating carcinogenic substances, such as, trihalomethanes (THMs) and haloacetic acids (HAAs) [6,7]. Furthermore, the presence of macromolecular dissolved organic materials may reduce the adsorption rates and equilibrium capacities of employed membranes or micro-porous adsorbents for water treatment processes [8-10]. For the treatment of humic acid-containing wastewater, various biological, physical and chemical methods such as microbial biodegradation, coagulation, filtration, ozonation, adsorption and heterogeneous photocatalysis have been used [11-16].

**Figure 1:**
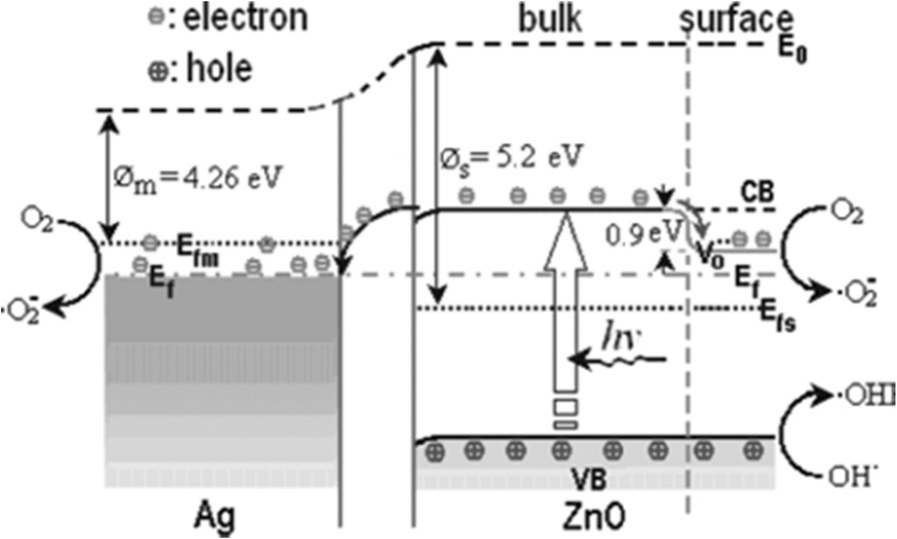
**Proposed band structure and photo-catalytic mechanism of theAg/ZnO under** ***h*** **υ- illumination** **[** 25 **].**

Among the methods for decreasing of organic pollutants, advanced oxidation processes (AOPs) has been considerable interest for the complete degradation many organic pollutants [17]. When a photocatalyst absorbs radiation whose energy hν > Eg (Eg is the semiconductor band gap energy), an ē from its filled valance band (VB) is promoted to its conduction band (CB) and valance band holes h^+^ are formed. Electron would reduce any available species, including O_2_, water and hydroxide ion to form hydroxyl radicals. The OH•^**¯**^radicals are very strong oxidizing agents and can easily attack the organic molecules, Thus leading finally to their complete mineralization. Among the various semiconductors employed as photocatalyst, TiO_2_ and ZnO are the most preferable material due to their non-toxic, insoluble, stability and high photoactivity properties [18-22]. Recently semiconductor-based hetero-structures have been attracted, due to their potential applications in various fields. Metal/semiconductor is one of the most popular heterostructures and has been extensively studied because of its excellent catalytic activity. Ag/ZnO is one of the heterostructure photocatalyst with high catalytic activity [23-26] that has attracted much research attention. Figure 1 shows the proposed band structure and photocatalytic mechanism of the Ag/ZnO and the photocatalytic reaction process (Eq. 1, 2, 3, 4, 5) was proposed by Zheng et al. [25]. Although Ag/ZnO was used to decolourization of several dyes [27,28] and photooxidation of humic acid on TiO_2_ and ZnO has been reported [14-16,29], in this research application of nano sized Ag/ZnO for photodegradation of humic acid is used for the first time in this paper.

1 $$ \mathrm{A}\mathrm{g}\to\ \mathrm{A}{\mathrm{g}}^{+}+{\mathrm{e}}^{-} $$

2 $$ \begin{array}{cc}\hfill {\mathrm{e}}^{-}+{\mathrm{O}}_2\to \hfill & \hfill {}^{.}{\mathrm{O}}_2^{-}\hfill \end{array} $$

3 $$ \mathrm{Z}\mathrm{n}\mathrm{O}+hv\to {\mathrm{e}}_{\mathrm{cb}}^{-}+{\mathrm{h}}_{\mathrm{vb}}^{+} $$

4 $$ {\mathrm{e}}_{\mathrm{cb}}+\mathrm{A}{\mathrm{g}}^{+}\to \mathrm{A}\mathrm{g} $$

5 $$ {\mathrm{h}}_{\mathrm{vb}}^{+}+\mathrm{O}{\mathrm{H}}^{-}{\to}^{.}\mathrm{O}\mathrm{H} $$

## Methods

### Reagents

All purchased chemicals were of reagent grade and used without further purification. Humic Acids (HA) was purchased from Sigma-Aldrich (Switzerland) and used without further purification.

### Apparatus

A Shimadzu Model 160-A UV–vis spectrophotometer with 10 mm quartz cells was used to make absorbance measurements. A Wegtech pH meter (Mi 151 22, England) was used for pH controlling was used for stirring the solutions. X-ray powder diffraction (XRD) measurements were performed using a Bruker, Advance D8 with Cu Kα (λ = 1.5406 Å) incident radiation. The size distribution and morphology of the synthesized catalyst was analyzed by scanning electron microscopy (SEM, Philips XL30) and transmission electron microscopy (Zeiss - EM10C - 80 KV).

### Preparation of Ag/ZnOnano structure

Zinc acetate (2 g) and silver acetate (0.2 g) were dissolved in water (80 mL). The pH value of the mixture was adjusted to 9 by triethyl amine. The reaction mixture was stirred for 30 min at room temperature. Then the reaction mixture was placed in a Parr-Teflon lined stainless steel vessel. It was sealed and heated at 130°C for 28 h. The reaction mixture was gradually cooled to room temperature. The resulting precipitate was filtered and washed using double distilled water.

### Photocatalytic degradation

Stock solution of the humic acid (50 mgL^−1^) was prepared by dissolving of 50 mg of humic acid in appropriate amount of NaOH 0.1 N and diluting to 1000 mL. Working solutions were prepared daily by diluting the stock solution with water.

HA degradation was performed in a batch type reactor and the temperature was kept at 25 + 2°C using a water circulator. In all cases during the experiment 50 mL of humic acid solution (25 mgL^−1^) containing the appropriate quantity of photocatalyst was magnetically stirred. In order to achieve the maximum adsorption of the sample onto the heterogonous photocatalyst, the cell was left for 20 min in the dark. The UV irradiation was carried out using Philips lamp (400 W). All photocatalytic experiments under solar irradiation were carried out under the similar conditions on sunny days of November between 11Am until noon. The colloid solutions of photocatalyst particles were centrifuged and the solution was separated. Decreasing in the concentration of humic acid was monitored spectrophotometrically by measuring the absorbance of dye at 254 nm [30]. All catalytic experiments were carried out at pH = 7 and for pH testing the pH of solution adjusted by 0.1 M sodium hydroxide or 0.1 M Hydrochloric acid. For The degree of photodegradation (X) as a function of time is given by Eq 6:

6 $$ X=\frac{{\mathrm{C}}_0-\mathrm{C}}{{\mathrm{C}}_0} $$

where C_0_ is the initial concentration of HA, and C the concentration of HA at time *t*. The light source emitted light just above the sample. The intensity level of light is controlled by fixing the distance between the source of light and the sample.

## Results and discussion

### Characterization of photocatalyst

**Figure 2:**
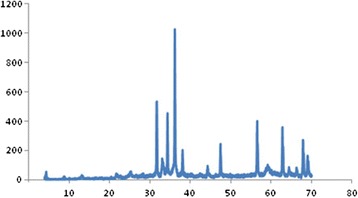
**The PXRD pattern of the synthesized nanosized Ag/ZnO.**

Figure 2 shows the XRD patterns of the Ag/ZnO catalyst. In Figure 2, three additional peaks at 36.18°, 48.36° and 68.58° compared to pure hexagonal-wurtzite ZnO (JCPDS Card Pattern: 36–1451), can be assigned to the face-centered cubic (fcc) structure of Ag crystallite (JCPDS Card Pattern 4–783), revealing that fcc structured Ag is produced in the reaction. The crystallite size is calculated Debye-Scherer formula, D = kλ/βcosθ where D is the crystallite size, k is a constant (= 0.9 assuming that the particles are spherical), λ is the wavelength of the X-ray radiation, β is the line width (obtained after correction for the instrumental broadening) and θ is the angle of diffraction. The average particle size obtained from XRD data is ~10 nm.

**Figure 3:**
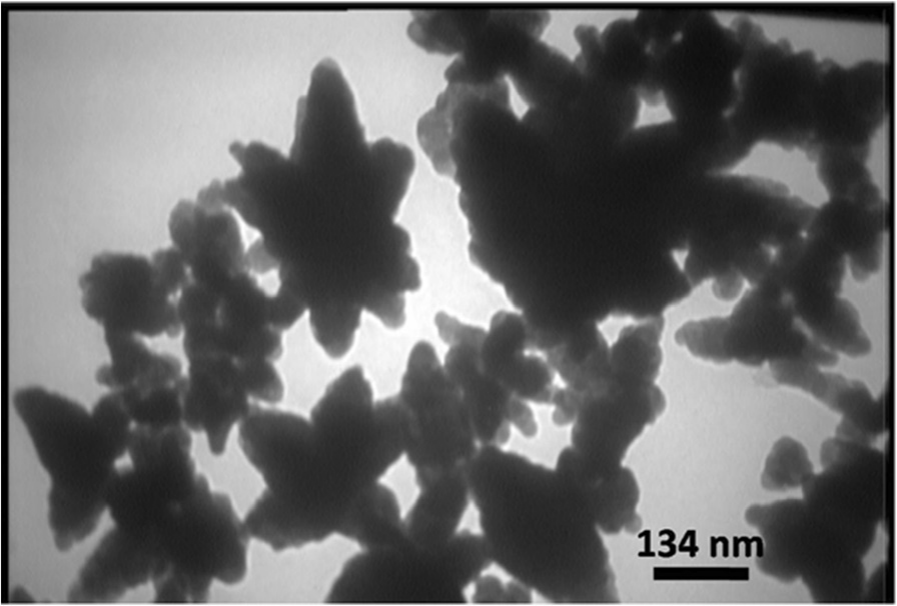
**The TEM image of the synthesized Ag/ZnO powder.**

The TEM micrograph (Figure 3) shows clearly that the nano-sized Ag/ZnO is in flower-like structure.

### Light effect

At first, experiments concerning the decomposition of HA (50 and 25 mgL^-1^) were performed, in the presence of Ag/ZnO semiconductor (0.5 g/L), UV_A_ and solar illumination, two blanks experiment in the absence of semiconductor and one blank in the absence of light. Results show that degradation of HA in the presence of the photocatalyst and irradiation could lead to the disappearance approximately 74% of HA under UV_A_ and 82% of HA under solar illumination after 40 min. Blank experiments in the absence of photocatalyst or light demonstrated no noticeable changes in the solution absorbance during of stirring of HA.

#### Effect of pH

**Figure 4:**
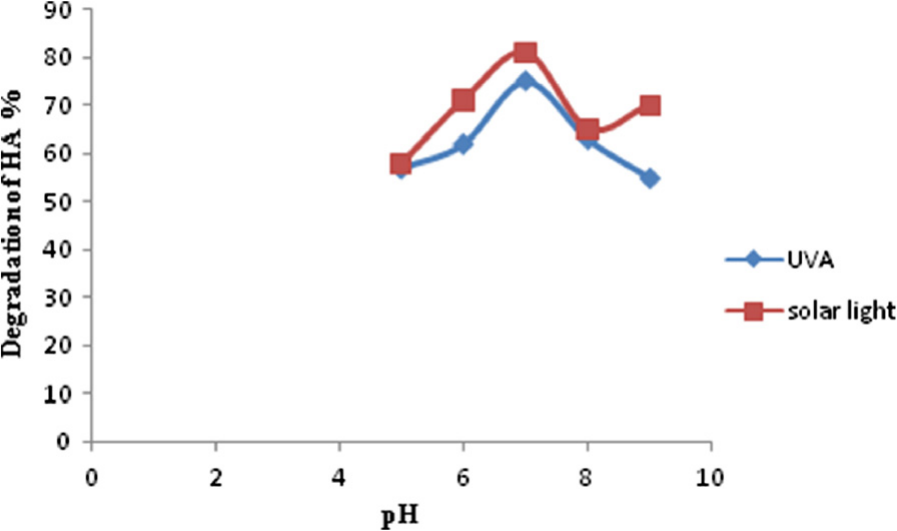
**Effect of pH on the photocatalytic efficiency percentages of HA on Ag/ZnOnano structure; Experimental conditions: HA = 25 mg/L, [Ag/ZnO] = 0.5 g/L, irradiation time = 40 min.**

The interpretation of pH effects on the efficiency of the photocatalytic degradation process is a very difficult. It depends to zero point charge of metal oxide and charge of pollution. Metal oxide surface is positively charged below zero point pH and above this negatively charged by adsorbed OH¯. The increasing of OH¯ concentration favors the formation OH• radical (principal oxidizing agent) and accelerates the oxidation reaction. But, it can decreases the electrostatic interactions between the metal oxide charge and pollution with negative charge [31]. The influence of pH on the photooxidation of humic acid was studied. The used humic acid is receipted in acidic solution. The initial pH of prepared HA is 10.4 and it decreases to 7.2 with addition of nano catalyst. Therefore, for the effect of pH on the degradation of HA by use of the Ag/ZnO, experiments were carried out at various pH ranging from 5 to 9 for constant HA concentration (25 mg/L) and catalyst amount of 0.5 g/L. Results show that faster degradation of HA was achieved at pH =7 (Figure 4).

#### Effect of catalyst concentration

**Figure 5:**
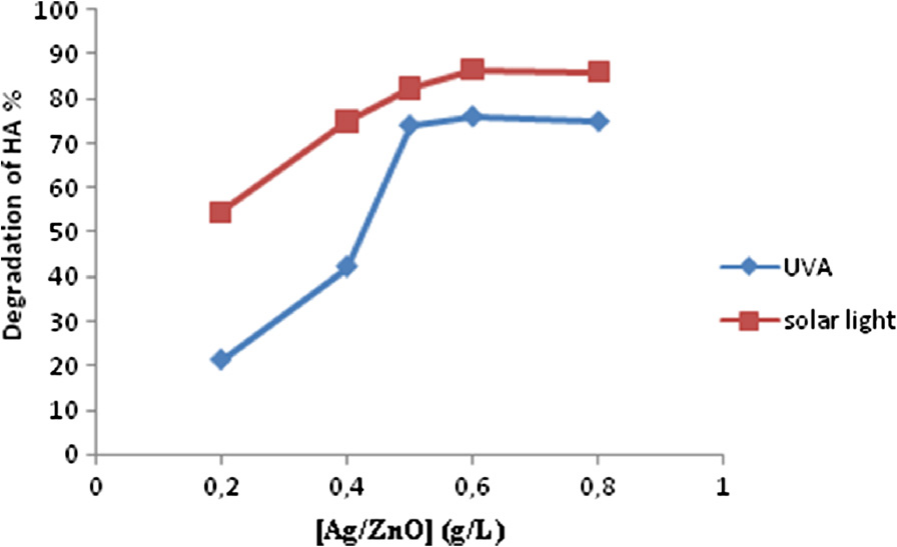
**Photodegradation of HA vs different Ag/ZnO concentration; Experimental conditions: HA = 25 mg/L, pH = 7, irradiation time = 40 min.**

In order to determine the effect of catalyst loading, the experiments were performed by varying catalyst concentration from 0.2 to 0.8 g/L for HA solution of 25 mg/L at pH = 7. The effect of the amount of Ag/ZnO on the photodegradation efficiency was shown in Figure 5. Experiments performed with different concentrations of synthesized Ag/ZnO reveals that initial slopes of the curves increase greatly by increasing catalyst loading from 0.2 to 0.6 g/L and thereafter the rate of degradation remains almost constant. This observation can be explained in terms of availability of active sites on the catalyst surface and the penetration of hυ light into the suspension. With increasing of catalyst dosage, total active sites on the catalyst surface increases, but the penetration of hυ light into the suspension may be decreases due to light scattering [18]. Therefore the catalyst dose 0.6 g/L were fixed for further studies.

#### Effect of concentration of humic acid

**Figure 6:**
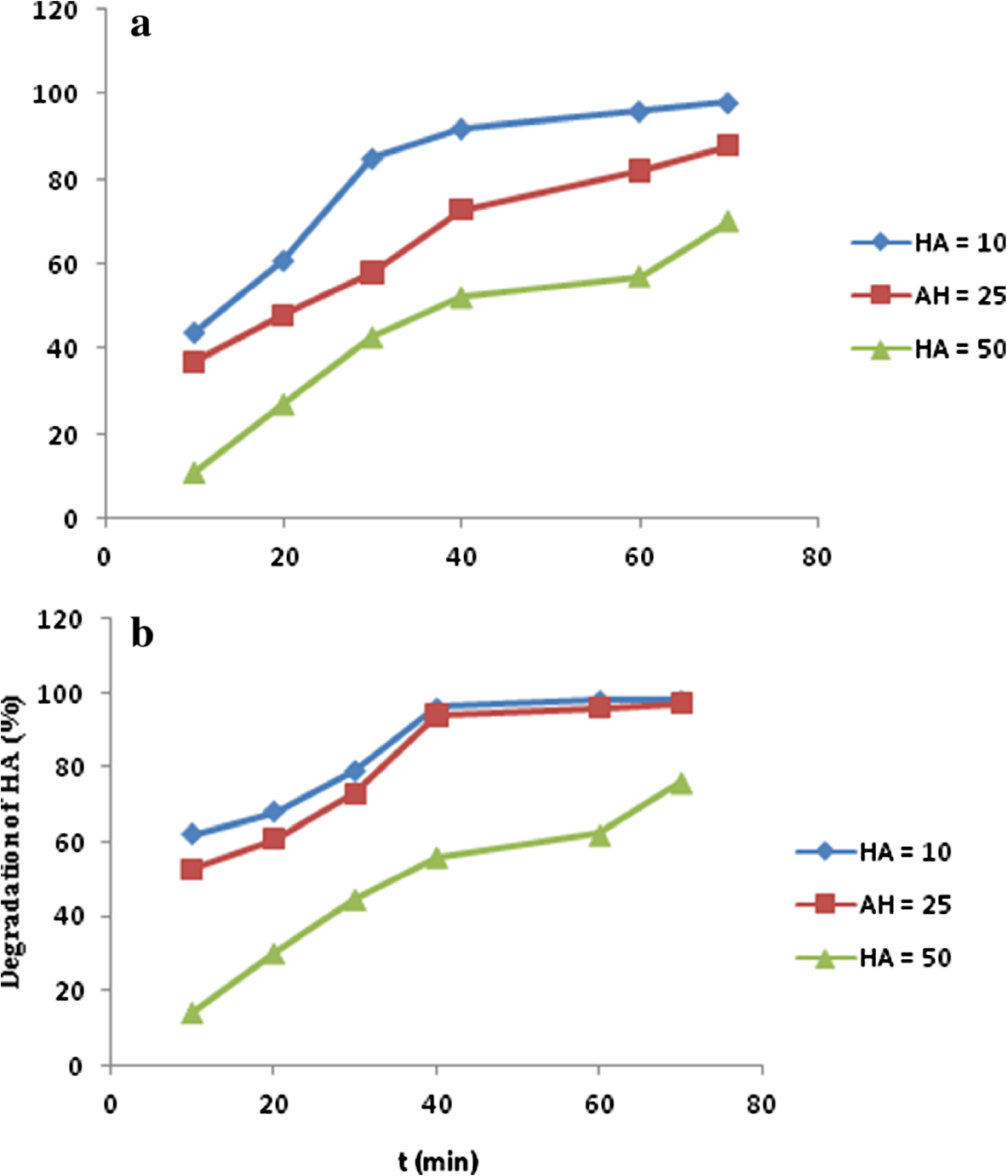
**Effect of the initial HA concentration on photocatalytic degradation of HA (pH = 7, Ag/ZnO = 0.6 g/L, a) UV**_**A**_ **irradiation and b) solar light).**

After optimizing the pH conditions and catalyst dose (pH 7.0 and catalyst dose o.6 g/L) the photocatalytic degradation of HA was carried out by varying the initial concentrations of the HA from 10 to 50 mg/L. Figure 6 shows the time dependent graphs of degradation of HA at different HA concentration solutions (10, 25 and 50 mg/L). As the concentration of the HA was increased, the rate of photodegradation decreased indicating to increase time scan for the complete degradation of HA. Faster degradation of HA was achieved under solar light for all cases.

For HA solution of 10 and 25 mg/L, more than 90% of pollution was degraded in the presence of solar light within 40 min and in case of 50 mg/L, almost 70% of degradation occurred within 70.

#### UV–vis spectra changes

**Figure 7:**
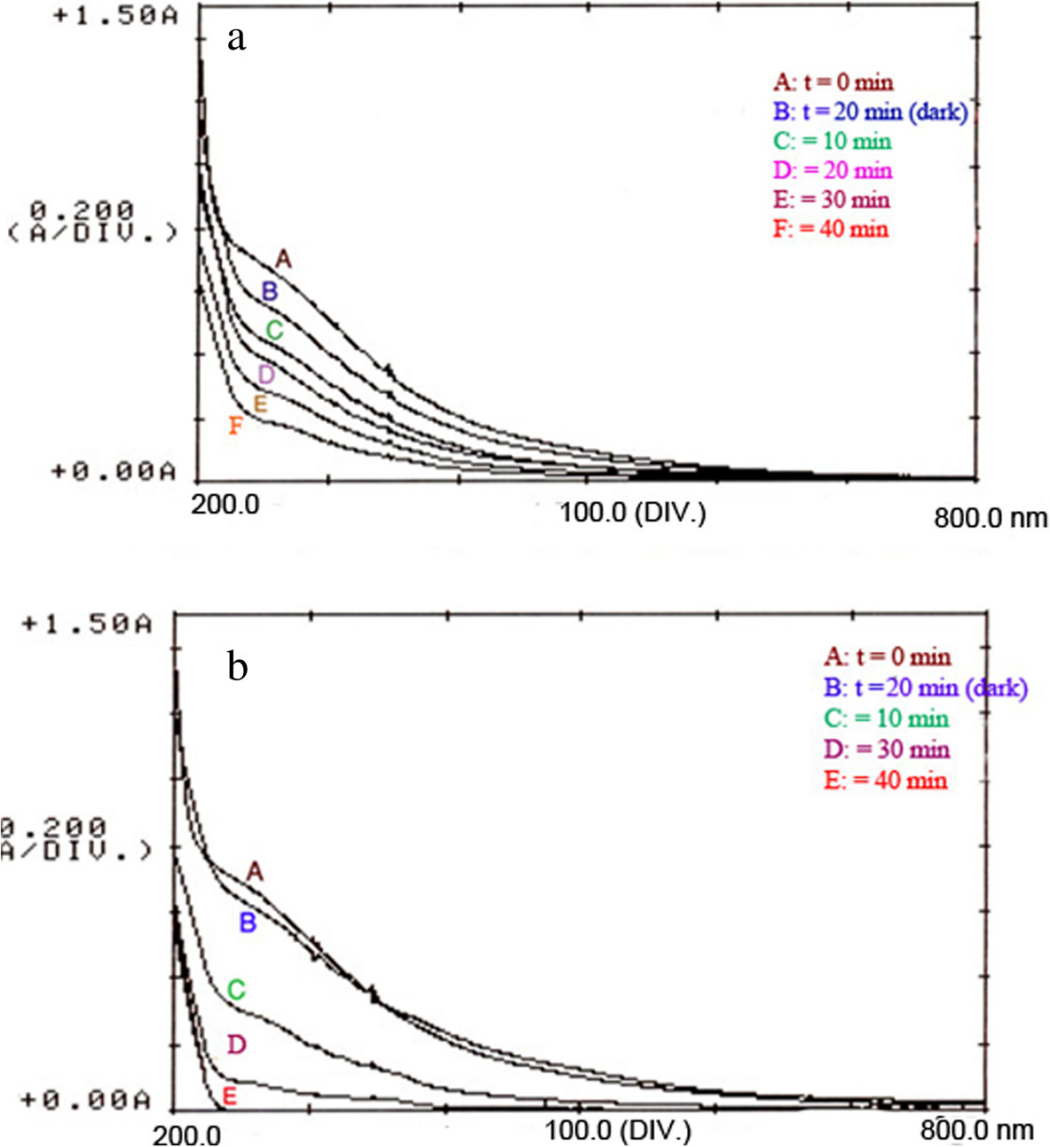
**UV–vis spectral changes of humic acid recorded during the HA degradation at different a) UVA b) solar irradiation times, (Ag/ZnO = 0.6 g/L; pH 7, HA = 25 mg/L).**

The changes in absorption spectra of humic acid during hν and Ag/ZnO process at different time irradiation intervals were shown in Figure 7. The decrease of the absorption band of HA at λ =254 nm indicates a rapid degradation of sample. Complete degradation of HA was observed after 40 minutes under solar irradiation in the optimized conditions. While, just 73% of HA degradation was done under UV_A_ irradiation.

#### Kinetic study

**Figure 8:**
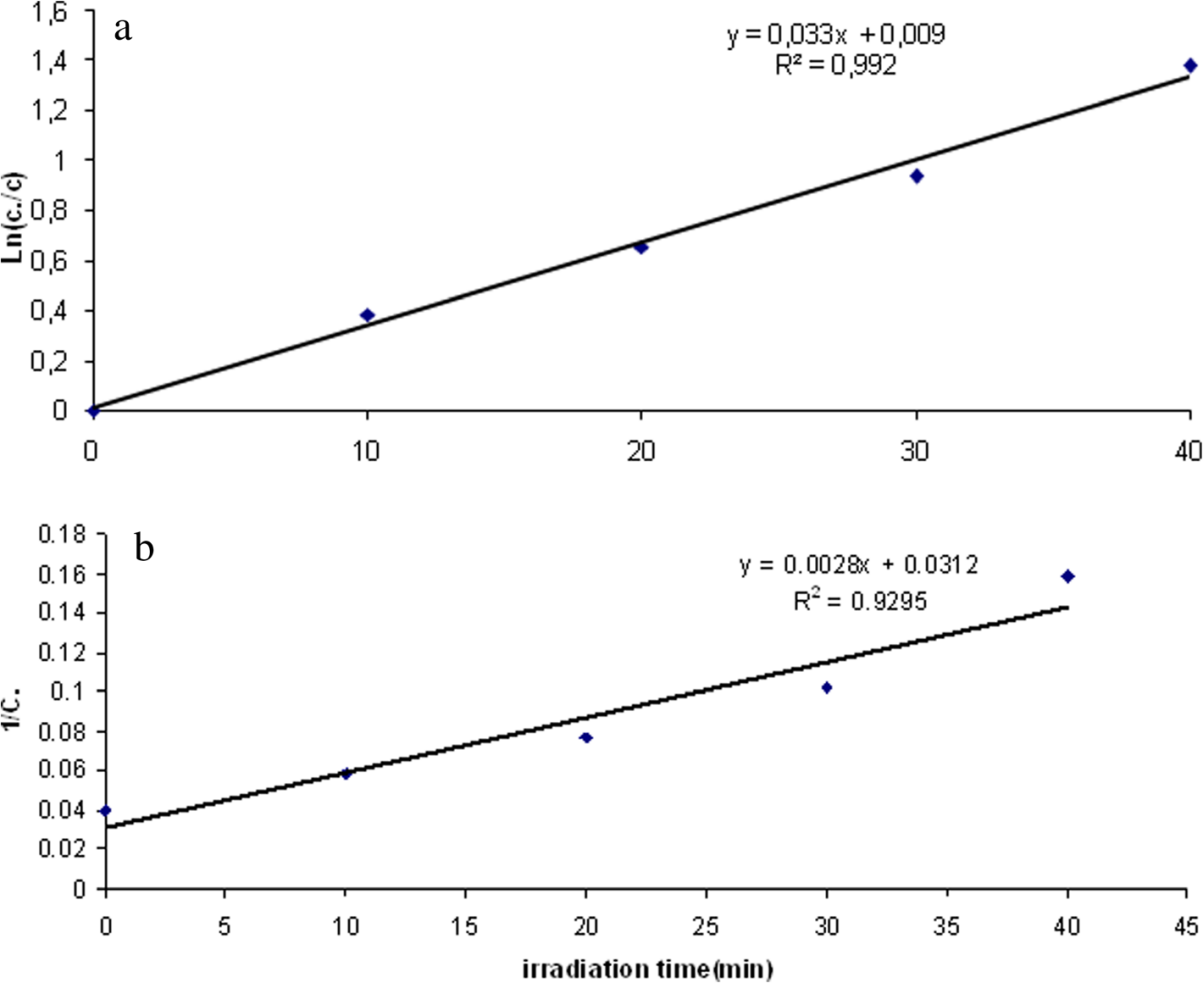
**The a) pseudo -first and b) pseudo -second order, kinetic models of photocatalytic degradation of HA under UV**_**A**_ **, (humic acid = 25 mg/L, pH = 7, Ag/ZnO = 0.6 g/L).**

**Figure 9:**
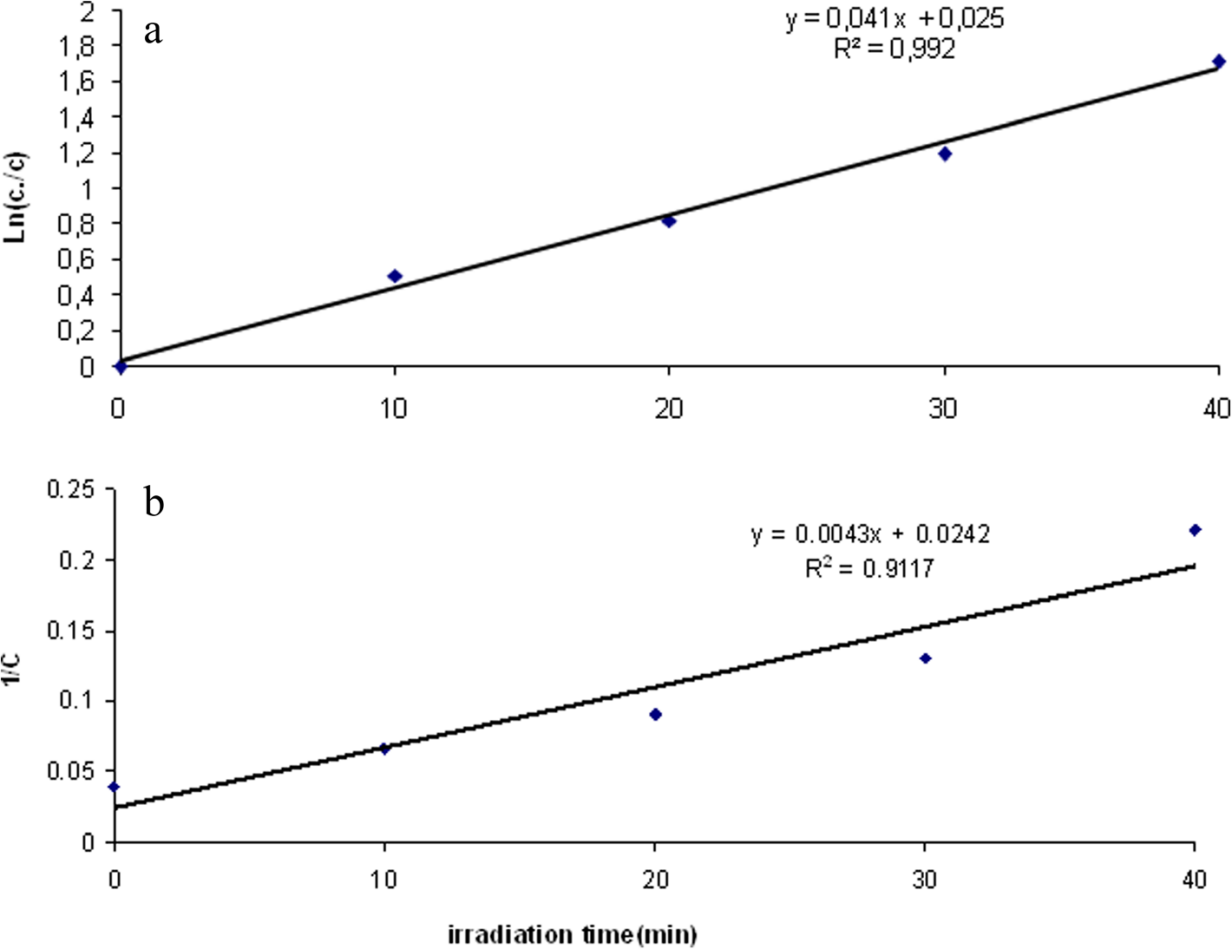
**The a) pseudo -first and b) pseudo -second order, kinetic models of photocatalytic degradation of HA under solar light, (humic acid = 25 mg/L, pH = 7, Ag/ZnO = 0.6 g/L).**

Kinetic models are used to examine the rate of the photocatalytic process and the potential rate controlling step. In the present work, the kinetic data obtained from batch studies have been analyzed using pseudo-first order and pseudo-second order models. For this purpose, 0.6 g/L of photocatalyst was used, reaction times were 10, 20, 30, 40 minutes and the pH of the solutions adjusted to 7. The results are shown in Figure 8a,b and Figure 9a,b under UVa and solar irradiation, respectively. According to these Figures, the correlation coefficient values (R^2^) of pseudo-first order were obtained 0.992 (Figures 8 and 9a)and 0.9295and 0.9117(Figures 8 and 9b) for second-order reactions. So, By comparison of those models, the correlation coefficients for the pseudo-first order kinetics model are higher than the correlation coefficients derived from pseudo-second order model fits. This suggests that the photodegradation process followed pseudo-first order kinetic.

## Conclusion

Nano-sized flower-like Ag/ZnO was synthesized and its photocatalytic effect on the degradation of humic acid under UV_A_ and solar irradiation was investigated. Flower-like Ag/ZnO nano structure is an efficient catalyst for the degradation rate of HA and degradation rate increased under solar light. Approximately 100% of HA has been eliminated after 40 minutes in the presences of catalyst and solar irradiation and without additional oxidation agent.
